# Effect of Bioflex crowns on periodontal health of primary molars indicated by interleukin-1 beta levels in gingival crevicular fluid: a randomized controlled clinical trial

**DOI:** 10.1186/s12903-026-10057-3

**Published:** 2026-11-03

**Authors:** Nadine Mahmoud Metwally, Magda M. El-Tekeya, Rasha Abdelmawla Ghazala, Marwa Baraka

**Affiliations:** 1 Pediatric Dentistry and Dental Public Health Department, Faculty of Dentistry, Alexandria University, Alexandria, Egypt; 2 Medical Biochemistry Department, Faculty of Medicine, Alexandria University, Alexandria, Egypt

**Keywords:** Stainless steel crown, Bioflex crown, Primary molars, Gingival health, Interleukin-1 beta, Gingival crevicular fluid

## Abstract

**Background:**

Stainless-steel crowns (SSCs) are durable and cost-effective, but do not fulfill aesthetic demands. This randomized controlled clinical trial compared the effects of Bioflex crowns and SSCs on the periodontal health of primary molars by measuring interleukin-1 beta (IL-1β) levels in the gingival crevicular fluid (GCF).

**Methods:**

A clinical split-mouth RCT was conducted on 20 children (4–8 years) who required full-coverage restorations on bilateral primary molars. In each child, one molar received a Bioflex crown, while the contralateral molar received an SSC. A third sound tooth served as an internal healthy control (total 60 teeth). GCF IL-1β concentration was measured at baseline and 7 and 90 days post-cementation using enzyme-linked immunosorbent assay (ELISA). Plaque Index (PI) and Gingival Index (GI) were recorded at all visits. Data were analyzed using generalized linear mixed models.

**Results:**

Baseline IL-1β levels were comparable (*p* > 0.05). At day 7, IL-1β levels significantly increased in the SSC group compared with the Bioflex and control groups (*p* < 0.001). By day 90, IL-1β levels in the SSC group decreased, and both SSC and Bioflex groups showed comparable results. No statistically significant differences in GI or PI scores were observed.

**Conclusions:**

Bioflex crowns demonstrated a more favorable early inflammatory response, indicated by the lower GCF IL-1β levels at day 7. By day 90, this initial inflammatory difference had resolved, and both crowns showed comparable periodontal health. These findings suggest that Bioflex crowns offer an esthetic full-coverage restorative option for primary molars without adversely affecting periodontal health.

**Trial registration:**

Pan African Clinical Trial Registry (www.pactr.samrc.ac.za; #PACTR202511815030369). Retrospectively registered on 17 November 2025.

**Supplementary Information:**

The online version contains supplementary material available at https://doi.org/10.1186/s12903-026-10057-3.

## Background

Preservation of the primary dentition is essential for children’s growth and development [1]. Severely decayed primary teeth present a challenge for pediatric dentists, requiring a balance between tooth preservation, restoration durability, and parental satisfaction [2]. The American Academy of Pediatric Dentistry recommends full-coverage restorations for posterior primary teeth with extensive decay, cervical decalcification, and developmental defects, following pulp treatment, and as definitive restorations in high caries–risk children [3].

Stainless steel crowns (SSCs) are the gold standard for full-coverage restorations in pediatric dentistry due to their superior longevity and durability [4–6], but they often fail to meet the aesthetic demands of parents and children because of their metallic appearance [7, 8]. Furthermore, achieving optimal marginal adaptation with SSCs requires clinical modifications such as cutting, crimping, and trimming. These mechanical alterations introduce surface defects and roughness, creating niches for microbial adhesion and plaque accumulation, potentially affecting periodontal health [9].

To address these aesthetic drawbacks, pre-veneered SSCs and zirconia crowns were introduced [4, 10]. Pre-veneered SSCs often exhibited porcelain chipping and margin exposure, which led to adverse gingival reactions [11, 12]. Conversely, zirconia crowns have demonstrated excellent aesthetics and biocompatibility, but they are rigid and require extensive tooth reduction, increasing the risk of pulp exposure [13, 14].

In 2022, NuSmile introduced the Bioflex crown as an alternative full-coverage restoration aimed at improving aesthetics while maintaining durability. Similar to SSCs, Bioflex crowns are pre-contoured and require comparable tooth preparation. They are composed of a high-impact hybrid resin–polymer free of bisphenol A–glycidyl methacrylate (BPA-GMA), offering a biocompatible and metal-free alternative [15, 16].

A fundamental objective of full-coverage restorations is periodontal health preservation. Prolonged contact between restorative materials and gingival tissue may disrupt local flora, causing plaque-induced inflammation and possible alveolar bone resorption [2]. Conventional diagnostic methods, including the Gingival Index (GI) and Plaque Index (PI), rely on visual indicators, such as color changes and bleeding upon probing. However, these indices are retrospective and subjective, failing to predict disease progression or inflammatory changes [17, 18].

The gingival crevicular fluid (GCF) serves as a sensitive, site-specific diagnostic tool. In the healthy state, the GCF is a physiologic transudate, but during inflammation, it transforms into an exudate rich in host mediators, including potent pro-inflammatory cytokines, such as interleukin-1 beta (IL-1β) [18, 19], which plays a central role in immune regulation and bone resorption [20, 21]. The GCF IL-1β level is highly correlated with periodontal challenge severity [22]. Notably, IL-1β levels have been shown to rapidly spike following plaque accumulation, often preceding visible clinical signs of inflammation, making IL-1β a vital early biomarker for assessing gingival inflammatory changes [23].

To date, no clinical randomized controlled trials (RCTs) have investigated the effect of Bioflex crowns on the periodontal health of primary molars using IL-1β as a biomarker. Understanding how these crowns affect the inflammatory response of the surrounding gingival tissue is essential for validating their biological safety and long-term clinical performance. Therefore, this clinical trial was designed to evaluate and compare the effect of Bioflex crowns and SSCs on periodontal health in restored primary molars. The primary aim was to assess the concentration of GCF IL-1β as a biomarker of marginal gingival inflammation in pediatric patients. The secondary aims were to clinically assess gingival health using the Modified Plaque Index (PI) and Gingival Index (GI).

The null hypothesis of this study was that no statistically significant difference exists between the periodontal and gingival health of primary molars restored with Bioflex crowns or SSCs.

## Materials and methods

### Ethical approval and study registration

The study was approved by the Ethical Committee of the Faculty of Dentistry, Alexandria University, Egypt (0890-03/2024; IRB #00010556-IORG:0008839), prior to participant recruitment. However, registration was completed retrospectively in the Pan African Clinical Trials Registry (PACTR) (www.pactr.samrc.ac.za; #PACTR202511815030369) on 17 November 2025 due to an administrative delay in completing the registration process. The study protocol, including the prespecified outcomes and statistical analysis plan, was finalized before recruitment began, and no changes were made after enrollment commenced. All procedures complied with the ethical standards of the 1975 Declaration of Helsinki, as revised in 2000 [24]. Written informed consent was obtained from the parents or legal guardians, after explaining the procedures and potential effects, and verbal assent was obtained from the children themselves.

### Study design and setting

**Fig. 1:**
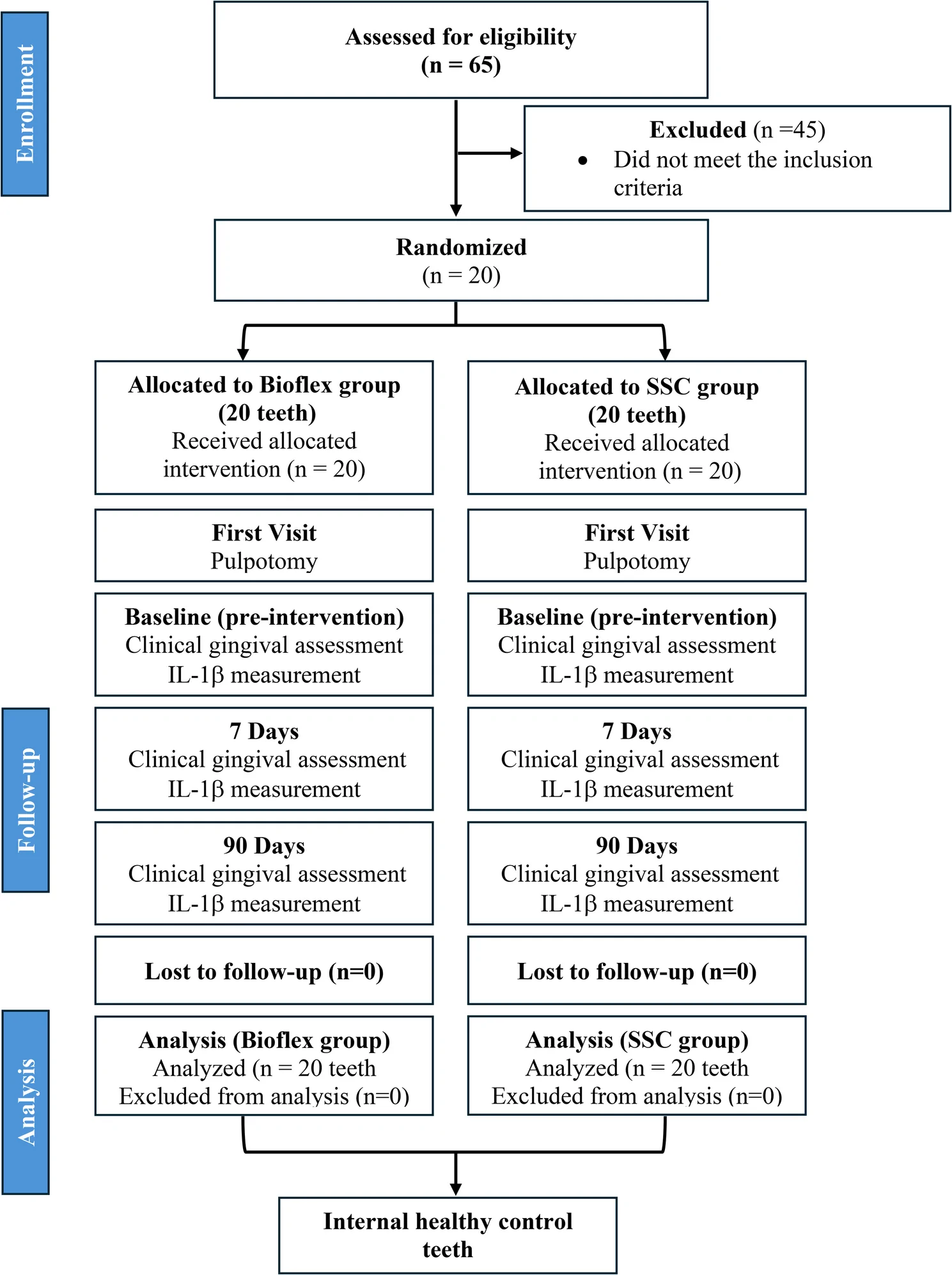
CONSORT flow diagram for the study. CONSORT: Consolidated Standards of Reporting Trials; IL-1β: interleukin-1 beta; SSC: stainless steel crown

This study was designed as a randomized, split-mouth controlled clinical trial, with a 1:1 allocation ratio. Clinical procedures were conducted at the Department of Pediatric Dentistry and Dental Public Health, Faculty of Dentistry, Alexandria University. Biochemical analysis was performed at the Medical Biochemistry Department, Faculty of Medicine, Alexandria University. All clinical procedures were performed by a single operator to eliminate interoperator variability. The Consolidated Standards of Reporting Trials (CONSORT) 2025 checklist was used for study design, analysis, and interpretation [25]. See Fig. 1 for a CONSORT flow diagram.

The PICOT question was: Among children aged 4–8 years with carious primary molars (P), did restoration with Bioflex crowns (I), compared with stainless-steel crowns (C), result in similar levels of interleukin-1β in the gingival crevicular fluid (O) after a 90-day follow-up period (T)?

### Sample size calculation

The sample size was estimated assuming a 95% confidence level and 80% study power. On the basis of the previous literature, compared to healthy teeth, the mean difference in IL-1β levels (primary outcome) was 3.74 for SSCs and 1.63 for zirconia crowns [2]. Because of their aesthetic and biocompatible properties, Bioflex crowns were hypothesized to yield results comparable to those of zirconia crowns [26]. Using a pooled standard deviation of 2.82, an assumed within-child correlation of 0.5 [2] and a calculated effect size of 0.748, a minimum sample size of 17 patients was required. To account for potential 10% loss to follow-up, we set the total enrollment at 20 patients using a split-mouth design. Calculations were performed using G*Power version 3.0.10 [27] based on Rosner’s method [28].

### Participant recruitment and eligibility

Participants were recruited from the Pediatric Dentistry Department’s outpatient clinic between January 5 and October 31, 2025. The study included 20 children aged 4–8 years requiring pulpotomies and full-coverage restorations on bilateral primary molars, with at least one sound tooth available to serve as an untreated healthy reference [2, 29].

The exclusion criteria were as follows:

- Children with generalized gingivitis or systemic diseases [2, 9, 30].
- Use of antibiotics within 4 weeks prior to or during the study [31].
- Patients with bruxism or noncompliance (Frankl Grade 1 or 2) [32, 33].
- Clinical signs of irreversible pulpitis (spontaneous or nocturnal pain), periapical abscesses, or abnormal tooth mobility [34].
- Radiographic evidence of widening of the periodontal ligament or furcal or periapical root resorption [34].
- Presence of proximal decay on the test teeth [35, 36].

### Randomization and blinding

Random allocation was used to determine which of the two eligible primary molars (right or left) received the Bioflex crown or the SSC, thereby assigning teeth to the Bioflex and SSC groups. Additionally, a sound tooth from the same child was included in the control group as an untreated healthy reference. For randomization, an assistant used a computer-generated list of random numbers (www.random.org). Allocation concealment was maintained using sequentially numbered, opaque envelopes, which were kept sealed and opened only at the time of the intervention. To minimize bias, our trial was single-blinded as the biochemist was blinded to the treatment groups. Blinding of the operator and patients was not possible due to the noticeable visual difference between the metallic SSCs and aesthetic Bioflex crowns.

### Examiner calibration

To ensure clinical diagnostic consistency and data reliability, a formal calibration phase was conducted prior to the commencement of the study. The investigator underwent calibration against a gold-standard examiner utilizing a pilot sample of 12 patients. For the GI, both the investigator and the examiner independently assessed the same patients on the same day. Fifteen standardized reference photographs were used for the PI calibration to avoid physical disruption of the biofilm during repeated clinical examinations [37]. Reliability for all clinical indices was confirmed after achieving Cohen’s Kappa of 0.82 [38].

### Intervention

#### First visit

During the initial visit, all participants received standardized oral hygiene instructions and dietary counseling [39]. Under rubber dam isolation, pulpotomies were performed on the indicated primary molars [40]. The molars were then restored with a glass ionomer cement (GIC) base (Fuji II LC; GC Corporation, Tokyo, Japan). To ensure that any localized inflammation resulting from the pulpotomy subsided before the baseline assessment, a stabilization period of 10 days was allowed, based on previous studies indicating that postoperative inflammatory changes following pulpotomy generally subside within this time frame [29].

#### Second visit (crown placement visit)

During the second visit, baseline biochemical and clinical assessments were performed. The GCF was collected first to prevent contamination, followed by PI and GI assessments.

### GCF sampling

For the biochemical assessment, the GCF was collected from the buccal sulcus of the primary molars and control teeth following a standardized protocol. To account for the daytime variation of GCF IL-1β, all clinical visits and sample collections were standardized and performed between 11:00 AM and 1:00 PM during the university’s clinical hours. Patients first rinsed their mouths with distilled water. Next, the sampling sites were isolated with cotton rolls and gently dried with air. Then, size 30 standardized paper points were inserted into the gingival crevice until resistance was felt and held for 30 s. To ensure sample purity, any paper points contaminated with blood or saliva were immediately discarded. Samples were placed in Eppendorf tubes containing 500 µL of phosphate-buffered saline and stored at − 80 °C until analysis via enzyme-linked immunosorbent assay (ELISA).

### Plaque and gingival indices

Following GCF collection, the Plaque Index (PI) was recorded according to Silness and Löe [41], , and the Gingival Index (GI) was recorded according to Löe and Silness [42]. Assessment involved four surfaces (mesial, distal, buccal, and lingual), and the average score per tooth was calculated. The PI was graded from 0 (no plaque) to 3 (abundance of plaque), and the GI was also graded from 0 (no inflammation) to 3 (severe inflammation).

### Crown placement

Following baseline measurements, the restorative procedures were carried out. Bioflex crowns and SSCs were obtained from NuSmile (Houston, TX, USA). All preparations were conducted according to the manufacturer’s instructions. When necessary, SSCs were trimmed and crimped with contouring and crimping pliers to achieve precise marginal adaptation. Both crown types were cemented using a self-cure GIC.

### Measurement of GCF IL-1β levels

Prior to analysis, samples were thawed and then centrifuged for 15 min at 1000×g at 2–8 °C. The samples were then analyzed using commercially available Human IL-1β ELISA kits (Catalog No. EH0185; FineTest Co., Ltd., Wuhan, China) according to the manufacturer’s instructions. Samples were analyzed undiluted, with each sample analyzed once without technical repetition. All samples obtained from the same child across the three assessment time points were analyzed on the same plate. According to the manufacturer, the intra-assay and inter-assay coefficients of variation ranged from 4.35% to 4.98% and 4.43% to 4.72%, respectively. Absorbance was read at a wavelength of 450 nm using a microplate reader (Human Diagnostics, Wiesbaden, Germany), and IL-1β concentrations were determined from a standard curve. The standard curve range for the assay was 3.906–250 pg/mL [43]. However, because the analytical sensitivity of the assay was 2.344 pg/mL, extrapolated sample values falling below the 3.906 pg/mL standard but above the sensitivity threshold were retained for statistical analysis without substitution.

### Follow-up and outcome assessment

Participants were called for follow-up visits at 7 and 90 days. During each follow-up, the GCF was collected to assess change in IL-1β levels, followed by PI and GI assessments. Oral hygiene was monitored, and oral hygiene instructions were reinforced at every visit to minimize confounding behavioral variables.

### Data analysis

Statistical analysis was performed using R (version 4.3.2 or later; R Foundation for Statistical Computing, Vienna, Austria). Data normality was evaluated using the Shapiro–Wilk test and visual inspection of Q–Q plots. A Generalized Linear Mixed Model (GLMM) was utilized to analyze the data within a repeated-measures, longitudinal design. To account for within-subject clustering across visits (Baseline, Day 7, and Day 90), Patient ID was included as a random intercept using an Unstructured (UN) covariance matrix and Restricted Maximum Likelihood (REML) parameter estimation. The fixed effects comprised Intervention (SSC, Bioflex, and Control), Time, and their two-way interaction (Intervention × Time). To accommodate the positive skewness of biochemical data, GCF IL-1β levels were modeled using a Gamma distribution with a log link function. Gingival and Plaque Indices were calculated as continuous subject-level mean scores across all examined sites and analyzed using a linear model (Gaussian distribution with identity link) with HC3 Robust Estimation (calculated via cluster-robust sandwich variance estimators to account for bounded scale constraints and heteroscedasticity). Model fit was evaluated using residual diagnostics (Q–Q and fitted vs. residual plots), which confirmed acceptable conditional normality and homoscedasticity. Significant main effects and interactions were further explored using Estimated Marginal Means, with pairwise comparisons adjusted using the Sequential Bonferroni method to control for family-wise Type I error. The level of statistical significance was set at *p* < 0.05.

## Results

### Participant characteristics

In total, 20 children (*n* = 9, 45%, males; *n* = 11, 55%, females) with a mean age of 6.10 ± 1.12 years were enrolled in this RCT. A total of 60 teeth were examined (SSC: *n* = 20, 33.3%; Bioflex: *n* = 20, 33.3%; control: *n* = 20, 33.3%). All participants completed the 90-day follow-up period.

Baseline GCF IL-1β concentrations and GI and PI scores did not differ significantly among groups (*p* > 0.05).

### IL-1β levels

The generalized linear mixed model (GLMM) revealed statistically significant effects of group (F = 29.57, *p* < 0.001), time (F = 28.98, *p* < 0.001), and the group × time interaction (F = 26.00, *p* < 0.001), indicating that IL-1β levels changed differently over time according to crown type.

At baseline, IL-1β levels were comparable among the three groups:

- SSC: 3.50 (95% CI: 2.55–4.45).
- Bioflex: 3.39 (95% CI: 2.44–4.33).
- Control: 3.48 (95% CI: 2.54–4.43).

At Day 7, a significant difference was observed between groups. IL-1β levels increased markedly in the SSC group to 11.15 (95% CI: 10.20–12.10), whereas the Bioflex and control groups remained stable at 3.62 (95% CI: 2.67–4.56) and 3.68 (95% CI: 2.73–4.63), respectively.

Pairwise comparisons showed:

- SSC vs. Bioflex: Mean difference = 7.54 (95% CI: 5.89–9.18), *p* < 0.001.
- SSC vs. Control: Mean difference = 7.47 (95% CI: 5.83–9.11), *p* < 0.001.
- Bioflex vs. Control: Mean difference = − 0.07 (95% CI: −1.71–1.58), *p* = 1.00.

By Day 90, IL-1β levels decreased in the SSC group to 3.81 (95% CI: 2.86–4.76), becoming comparable to Bioflex (3.56; 95% CI: 2.61–4.51) and control (3.59; 95% CI: 2.64–4.54). No statistically significant differences were detected between groups.

**Fig. 2:**
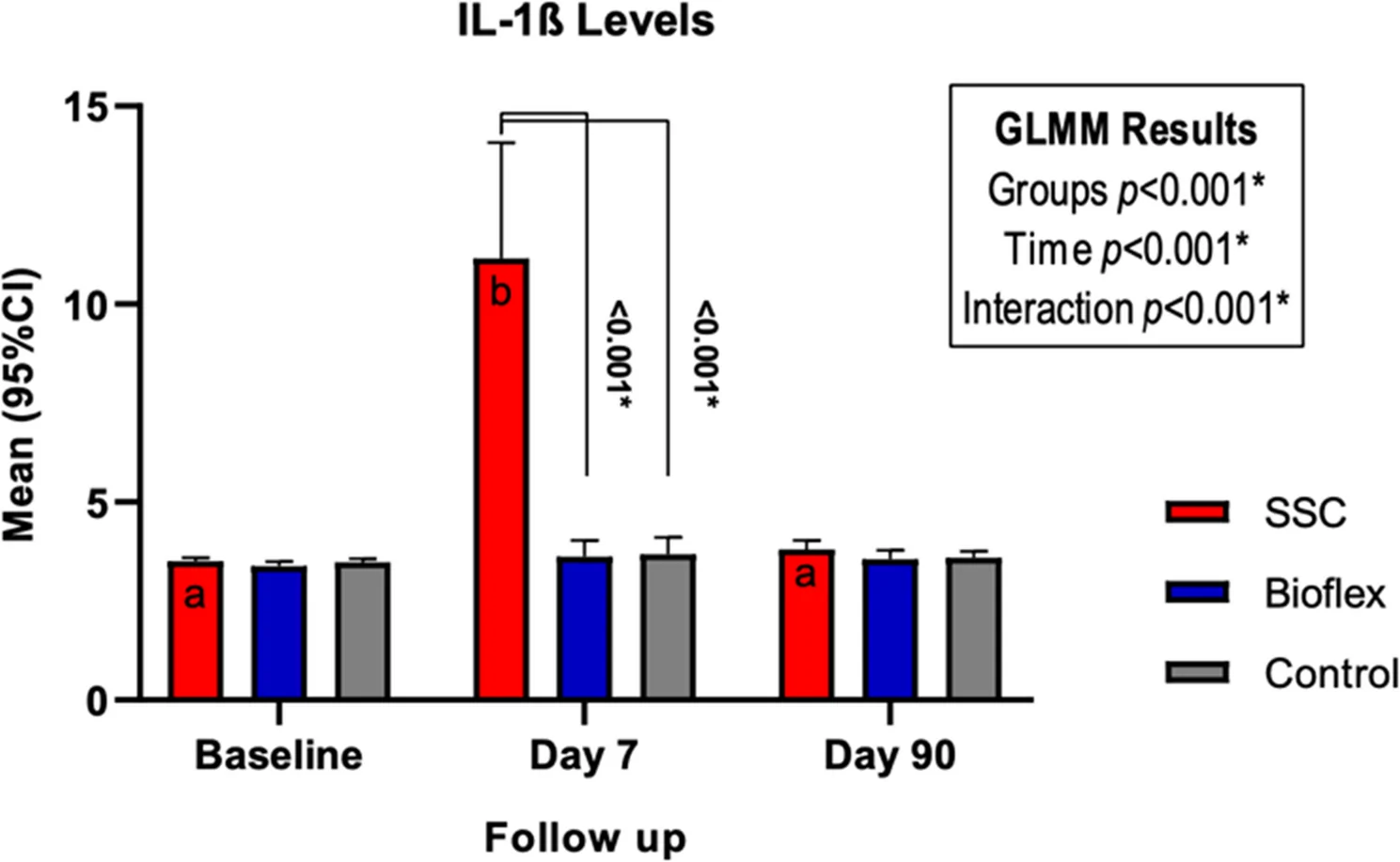
GLMM assessing the effect of interventions and time on GCF IL-1β levels. GCF: gingival crevicular fluid; GLMM: generalized linear mixed model; IL-1β: interleukin-1 beta; SSC: stainless steel crown

Within-group analysis showed a significant increase in IL-1β in the SSC group from baseline to Day 7 (mean difference = − 7.65, *p* < 0.001) and a significant decrease from Day 7 to Day 90 (mean difference = 7.34, *p* < 0.001), with no difference between baseline and Day 90 (*p* = 1.00). No significant changes over time were observed in the Bioflex or control groups (*p* = 1.00) ) (Fig. 2; Tables 1 and 2).

**Table 1:** Comparison of IL-1β between SSC and Bioflex crowns at different time intervals

Follow up		SSC	Bioflex	Control
Baseline	EM mean (95% CI)	3.50 (2.55, 4.45)^aA^	3.39 (2.44, 4.33)^aA^	3.48 (2.54, 4.43)^aA^
	Median (IQR)	3.50 (0.35)	3.30 (0.45)	3.50 (0.38)
Day 7	EM mean (95% CI)	11.15 (10.20, 12.10)^aB^	3.62 (2.67, 4.56)^bA^	3.68 (2.73, 4.63)^bA^
	Median (IQR)	10.50 (13.43)	3.40 (0.30)	3.50 (0.38)
Day 90	EM mean (95% CI)	3.81 (2.86, 4.76)^aA^	3.56 (2.61, 4.51)^aA^	3.59 (2.64, 4.54)^aA^
	Median (IQR)	3.75 (0.75)	3.40 (0.28)	3.40 (0.58)

Different superscript lowercase letters denote statistically significant differences between groups

*SSC* stainless steel crown, *EM* estimated marginal mean, *CI* confidence interval, *IQR* interquartile range

**Table 2:** Pairwise comparisons of IL-1β between groups within each time interval

Groups	Time	Compared to	Mean Difference	p value	95% Confidence Interval	
					Lower Bound	Upper Bound
Baseline	SSC	Bioflex	0.12	1.00	-1.53	Baseline
		Control	0.02	1.00	-1.63	1.66
	Bioflex	Control	-0.10	1.00	-1.74	1.54
Day 7	SSC	Bioflex	7.54	< 0.001*	5.89	Day 7
		Control	7.47	< 0.001*	5.83	9.11
	Bioflex	Control	-0.07	1.00	-1.71	1.58
Day 90	SSC	Bioflex	0.25	1.00	-1.39	Day 90
		Control	0.22	1.00	-1.42	1.86
	Bioflex	Control	-0.03	1.00	-1.67	1.61

*SSC* stainless steel crown

*Statistically significant difference at p-value < 0.05

### Gingival index

GLMM analysis showed no significant effect of group (*p* = 0.829), time (*p* = 0.297), or group × time interaction (*p* = 0.890) on GI scores throughout the study period (Table 3).

**Table 3:** Comparison of GI between SSC and Bioflex groups at different time intervals

Follow-up	Statistical Parameter	Bioflex group	SSC group	Control group
Baseline	EM mean (95% CI)	0.43 (0.22–0.64)	0.44 (0.31–0.57)	0.41 (0.27–0.55)
	Median (IQR)	0.38 (0.50)	0.50 (0.25)	0.38 (0.69)
Day 7	EM mean (95% CI)	0.43 (0.21–0.64)	0.45 (0.30–0.60)	0.41 (0.30–0.53)
	Median (IQR)	0.25 (1.00)	0.50 (0.63)	0.38 (0.25)
Day 90	EM mean (95% CI)	0.50 (0.30–0.70)	0.51 (0.34–0.68)	0.43 (0.30–0.55)
	Median (IQR)	0.50 (1.00)	0.50 (0.75)	0.38 (0.25)

*GI *Gingival Index, *SSC* stainless steel crown, *EM* estimated marginal mean, *CI* confidence interval, *IQR* interquartile range

### Plaque index

Similarly, GLMM analysis showed no significant effect of group (*p* = 0.957), time (*p* = 0.403), or group × time interaction (*p* = 0.992) on PI scores, at all follow-up intervals (Table 4).

**Table 4:** Comparison of PI between SSC and Bioflex groups at different time intervals

Follow-up	Statistical Parameter	Bioflex group	SSC group	Control group
Baseline	EM mean (95% CI)	0.40 (0.25–0.55)	0.41 (0.24–0.58)	0.39 (0.27–0.51)
	Median (IQR^e^)	0.50 (0.50)	0.25 (0.75)	0.25 (0.25)
Day 7	EM mean (95% CI)	0.43 (0.24–0.61)	0.43 (0.29–0.56)	0.41 (0.29–0.54)
	Median (IQR)	0.50 (0.50)	0.50 (0.69)	0.50 (0.44)
Day 90	EM mean (95% CI)	0.45 (0.27–0.63)	0.46 (0.35–0.57)	0.43 (0.28–0.57)
	Median (IQR)	0.50 (0.75)	0.50 (0.50)	0.38 (0.69)

*PI* Plaque Index, *SSC* stainless steel crown, *EM* estimated marginal mean, *CI* confidence interval, *IQR* interquartile range

## Discussion

Rising aesthetic expectations among parents and children have driven the development of prefabricated crowns from metallic, colored ones toward alternatives that more closely resemble natural teeth. These alternatives include zirconia crowns and the most recent resin polymer hybrid Bioflex crown [44]. Maintaining periodontal health is a primary goal of full-coverage restorations. However, differences in the composition of restorative materials and marginal adaptation may influence the gingival response [45], which could potentially result in inflammatory changes that are not immediately detectable through clinical examination [46].

Bioflex crowns were selected for this study because they are marketed as the first flexible, durable, and esthetic pediatric crown. They are manufactured from a biocompatible, high-impact resin material. Clinically, their tooth preparation requirements lie between those of Zirconia and SSCs [33]. To evaluate the periodontal response to this material, Interleukin-1 beta (IL-1β) was selected as a biomarker of marginal gingival inflammation, as elevated levels of IL-1β in gingival crevicular fluid (GCF) are strongly associated with periodontal inflammation and have been shown to correlate with disease severity [47]. To date, no studies have investigated the effect of Bioflex crowns on GCF IL-1β levels. Therefore, the primary objective of this clinical trial was to evaluate the local inflammatory response associated with Bioflex crowns compared with SSCs using IL-1β as a biochemical marker.

The results demonstrated that both crown types showed comparable outcomes in terms of IL-1β concentrations, plaque accumulation, and gingival health by day 90. However, a clear difference was observed at follow-up on day 7, where the GCF IL-1β levels exhibited a significant transient increase in the SSC group but remained stable in the Bioflex group (*p* < 0.001). This rise is biologically relevant, as IL-1β is a potent pro-inflammatory cytokine that recruits neutrophils to inflamed tissues and plays a central role in alveolar bone resorption and connective tissue breakdown [48].

The initial rise in GCF IL-1β levels associated with SSCs observed in our study is hypothesized to be multifactorial. Potential explanations may include their metallic composition, mechanical manipulation during crown adaptation, or procedural factors associated with crown placement such as residual subgingival glass ionomer cement. Consistent with findings by Aggarwal et al. (2022) and Gölz et al. (2014), the release of nickel and chromium ions from SSCs can act as “danger signals” capable of activating inflammatory signaling that stimulates IL-1β expression [2, 49]. Furthermore, they require trimming, cutting, and crimping for clinical use, which can cause surface irregularities and increase roughness, potentially creating niches that promote microbial adhesion and plaque accumulation [50]. Moreover, mechanical manipulation of the metal may lead to ion release at the gingival margin [51]. Collectively, these factors may explain the pronounced increase in GCF IL-1β levels observed in the SSC group.

Conversely, the absence of a transient inflammatory surge in the Bioflex group may be consistent with a potentially favorable biological response to this restoration. As Yadolahi et al. (2026) described, Bioflex crowns utilize a self-adapting hybrid polymer that mitigates the rigidity associated with traditional crowns, allowing their adaptation to molars without excessive manipulation. Additionally, they noted an absence of systemic adverse events or localized hypersensitivity with these restorations, attributing this to their metal-free composition [52]. Ultimately, the absence of metal ion leaching combined with reduced mechanical manipulation at the gingival margin may contribute to a favorable environment that effectively prevents early subclinical gingival inflammation.

Despite early molecular differences, no significant between-group differences were observed in plaque accumulation and gingival inflammation scores. These findings are aligned with previous research by Abdelhafez and Dhar (2025) and Singh et al. (2025), who found that gingival health was comparable between the two groups, with no significant differences [50, 53]. The discrepancy between clinical indices (GI and PI) and GCF IL-1β levels on day 7 can be explained by the biological behavior of inflammatory mediators. GCF IL-1β levels rise rapidly at the molecular level, often within 24 h to 7 days, preceding clinically detectable signs, such as erythema, edema, or bleeding on probing, which are reflected in the GI score (GI) [23].

By day 90, GCF IL-1β levels in the SSC group had returned to near-baseline values, achieving statistical equivalence with both Bioflex and control groups. This stabilization reflects the adaptation of the periodontal tissues to the restoration as the initial mechanical trauma associated with crown preparation and marginal adaptation subsides, and the leaching of these pro-inflammatory ions decreases, which peaks within the first 7 days and then gradually declines [53, 54]. With adequate oral hygiene, the tissue fully adapts once the initial healing phase is complete. Nevertheless, the transient inflammatory surge observed during postoperative week 1 remains clinically relevant: elevated pro-inflammatory cytokine levels during this period may contribute to postoperative discomfort or localized bone remodeling effects that may be mitigated by using more biocompatible materials, such as Bioflex.

The findings of our current study are consistent with a favorable short-term biological response to Bioflex crowns. As these crowns are made from a metal-free resin polymer, it is possible that their material characteristics may reduce exposure to metal ions associated with SSCs. However, ion release and other material-related mechanisms were not directly assessed in this study. Consequently, the data from this study suggest that Bioflex crowns are not associated with sustained adverse effects on gingival and periodontal health over the 90-day follow-up period. Although a statistically significant transient difference in GCF IL-1β levels was observed between Bioflex and stainless steel crowns at day 7, no significant differences in the clinical indices or biochemical parameters remained at the 90-day follow-up. Accordingly, the null hypothesis of no difference in GCF IL-1β levels between Bioflex and stainless steel crowns was rejected at day 7; however, the observed difference was not sustained at 90 days.

A significant strength of this study was the absence of loss to follow-up, achieved by reinforcing attendance through appointment day reminders. Moreover, a single trained pediatric dentist placed the crowns, minimizing interexaminer variability and enhancing the reliability and validity of the findings. Further, to reduce confounding factors, oral hygiene instructions were reinforced at every visit. This allowed plaque accumulation around the crowns to be assessed independently of oral hygiene practices. The split-mouth design further strengthened the study by using each patient as their own control, effectively eliminating individual variability related to systemic and immune responses, oral hygiene behavior, and genetic polymorphism [55, 56]. The inclusion of a sound tooth as a control site also confirmed that baseline GCF IL-1β levels remained stable throughout the study period, suggesting that the transient increase observed in GCF IL-1β levels at day 7 in the SSC group was attributable to the restorative process itself.

This study has a few limitations. First, we were unable to implement a double-blind design, as operator and patient blinding to the crown type was not feasible. Second, although the 7- and 90-day follow-up periods were adequate to capture the initial inflammatory response and subsequent stabilization [29, 57], an intermediate follow-up interval could provide additional insight into when GCF IL-1β levels begin to normalize. Additionally, longer follow-up periods can help to assess long-term clinical performance. Furthermore, although IL-1β served as a sensitive indicator of pro-inflammatory activity, periodontal inflammation involves a multifactorial network of mediators. Future investigations should incorporate a broad cytokine profile, including various markers, to better understand the host response to Bioflex crowns. Finally, although excess luting cement was carefully removed during crown placement according to routine clinical practice, no objective assessment was performed to verify complete cement removal, marginal adaptation, or the position of the crown margins relative to the gingival margin, and no radiographic assessment of residual subgingival cement was undertaken. Therefore, procedural factors, particularly marginal adaptation and residual subgingival cement, cannot be excluded as alternative explanations for the transient inflammatory response observed with SSCs. Future studies should incorporate standardized objective assessments of crown adaptation and margin position, together with appropriate methods for detecting residual subgingival cement, to help distinguish material-related effects from procedural influences on periodontal inflammation.

## Conclusion

In conclusion, compared to SSCs, Bioflex crowns show a more favorable early periodontal response, evidenced by the lower GCF IL-1β levels after week 1. This early response in the SSC group was transient and resolved by the 90-day follow-up, with both SSC and Bioflex groups demonstrating comparable periodontal health. Clinical indices of plaque accumulation (PI) and gingival inflammation (GI) did not differ significantly between groups. This suggests that Bioflex crowns provide an aesthetic and metal-free alternative to SSCs without adversely affecting periodontal health in primary molars.

## Supplementary Information

Below is the link to the electronic supplementary material.

Supplementary Material 1.

Supplementary Material 2.

Supplementary Material 3.

## Data Availability

All data included in this study are available from the corresponding author upon request.

## Abbreviations

BPA-GMA: Bisphenol A-glycidyl methacrylate
ELISA: Enzyme-Linked Immunosorbent Assay
GCF: Gingival Crevicular Fluid
GI: Gingival Index
GLMM: Generalized Linear Mixed Model
GIC: Glass Ionomer Cement
IL-1β: Interleukin-1 Beta
PBS: Phosphate-Buffered Saline
PDL: Periodontal Ligament
PI: Plaque Index
RCT: Randomized Controlled Clinical Trial
SSC / SSCs: Stainless Steel Crown / Stainless Steel Crowns
