# Supplementary material for: Effect of Bioflex crowns on periodontal health of primary molars indicated by interleukin-1 beta levels in gingival crevicular fluid: a randomized controlled clinical trial
Source: BMC Oral Health. 2026 Nov 3;26:2213. doi: 10.1186/s12903-026-10057-3 (PMC13746536; doi:10.1186/s12903-026-10057-3)
Supplement: Supplementary file 2 [file 12903_2026_10057_MOESM2_ESM.pdf]

IRB NO: 00010556 – IORG 0008839
https://ohrp.cit.nih.gov/search/search.aspx

جامعة الإسكندرية
ALEXANDRIA
UNIVERSITY
كلية طب الاسنان
FACULTY OF DENISTRY
كليـــه معتمـــدة

Alex: 4/11/2025

Statement

The Scientific Research Ethics Committee at the Faculty of Dentistry, Alexandria University, certifies the approval of the scientific research submitted by the dentist -: **Nadine Mahmoud Mohamed Metwally in Pediatric Dentistry,on 18/03/2024.**

Research Title:

EFFECT OF BIOFLEX CROWNS ON THE PERIODONTAL HEALTH OF PRIMARY MOLARS INDICATED BY INTERLEUKIN-ONE BETA CONTENT OF GINGIVAL CREVICULAR FLUID A RANDOMIZED CONTROLLED CLINICAL TRIAL

SCCREIRB-DNTSTRYALX-GU-001-180325-042
IRB by OHRP: 00010556
IORG: 0008839
Ethics Committee No: 0890 – 03/2024.
This is a statement from us to be submitted To Whom It May Concern.

Vice Dean for
Graduate Studies & Research

Riham Eldiba

Prof. Dr. Riham Mostafa EL-Dibany

Scanned with CamScanner
